# Supplementary material for: Flat-top TIRF illumination boosts DNA-PAINT imaging and quantification
Source: Nat Commun. 2019 Mar 20;10:1268. doi: 10.1038/s41467-019-09064-6 (PMC6426843; doi:10.1038/s41467-019-09064-6)
Supplement: Supplementary file 1 — Supplementary Information [file 41467_2019_9064_MOESM1_ESM.pdf]

Supplementary Information

Flat-top TIRF illumination boosts DNA-PAINT imaging and quantification

Stehr, Stein et al.

**Supplementary Figure 1**	Schematic of custom-built super-resolution microscopy setup
**Supplementary Figure 2**	Spot detection and net gradient
**Supplementary Figure 3**	Bright time distributions for 8-nt vs. 9-nt docking site length
**Supplementary Figure 4**	Averaged 20-nm-grid image for 8-nt vs. 9-nt docking sites length for flat-top excitation
**Supplementary Figure 5**	Individual origami images for averaging
**Supplementary Figure 6**	Segment-wise photon count distribution Gauss vs. Flat-top
**Supplementary Figure 7**	Uniform spot-detection efficiency for cellular DNA-PAINT imaging
**Supplementary Figure 8**	Mislocalization removal by multi-emitter filtering
**Supplementary Figure 9**	Mislocalization removal by multi-emitter filtering for Gaussian illumination
**Supplementary Figure 10**	DNA origami grid designs
**Supplementary Figure 11**	Laser power comparison Gauss vs. Flat-Top
**Supplementary Figure 12**	Filtering by temporal distribution of localizations
**Supplementary Table 1**	List of core staples
**Supplementary Table 2**	List of biotinylated staples
**Supplementary Table 3**	Imaging parameters
**Supplementary Table 4**	DNA-PAINT imager sequences

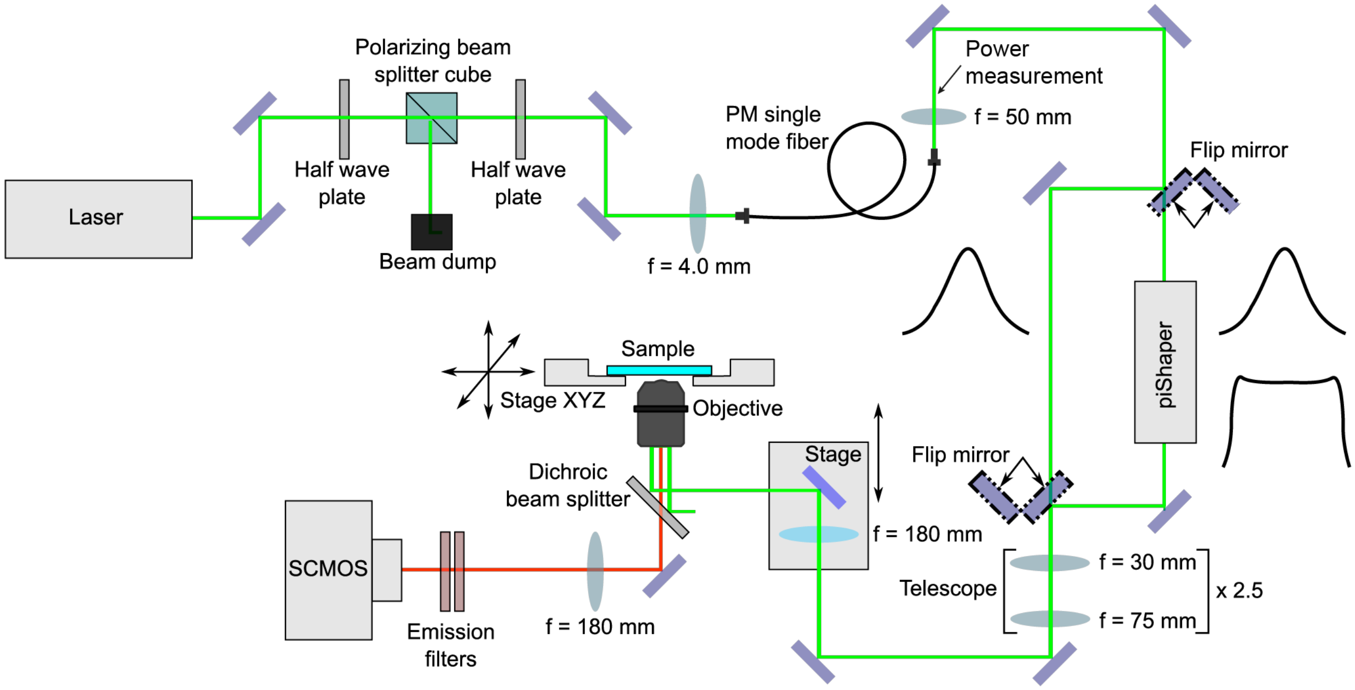

Supplementary Figure 1 | Schematic of custom-built super-resolution microscopy setup. Blue rectangles indicate dielectric mirrors, ellipses indicate lenses.

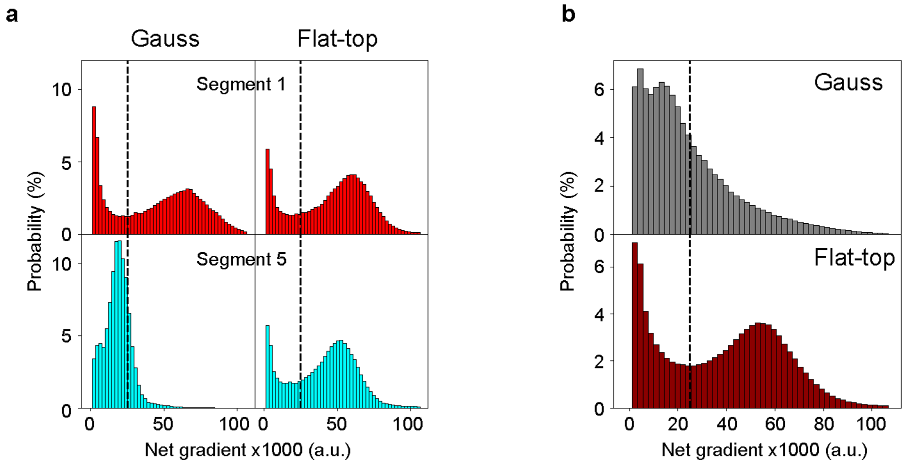

Supplementary Figure 2 | Spot detection and net gradient. a Net gradient histograms comparing segments 1 and 5 of 20-nm-grid images acquired with Gaussian and flat-top illumination. For DNA-PAINT (but also for SMLM in general) a localization algorithm implemented in Picasso[1] distinguishes between bright blinking events and background noise by computing the net gradient between adjacent pixels in the raw images. A threshold value (dashed line) is chosen manually such that in the center (segment 1) only blinking events and no background is recognized. Due to the inhomogeneous illumination of a Gaussian profile the net gradient in segment 5 does not reach this threshold anymore and blinking events are not recognized. DNA-PAINT data acquired under flat-top illumination show a homogeneous net gradient comparing segments 1 and 5. **b** Net gradient sum histograms for the two images from **a**

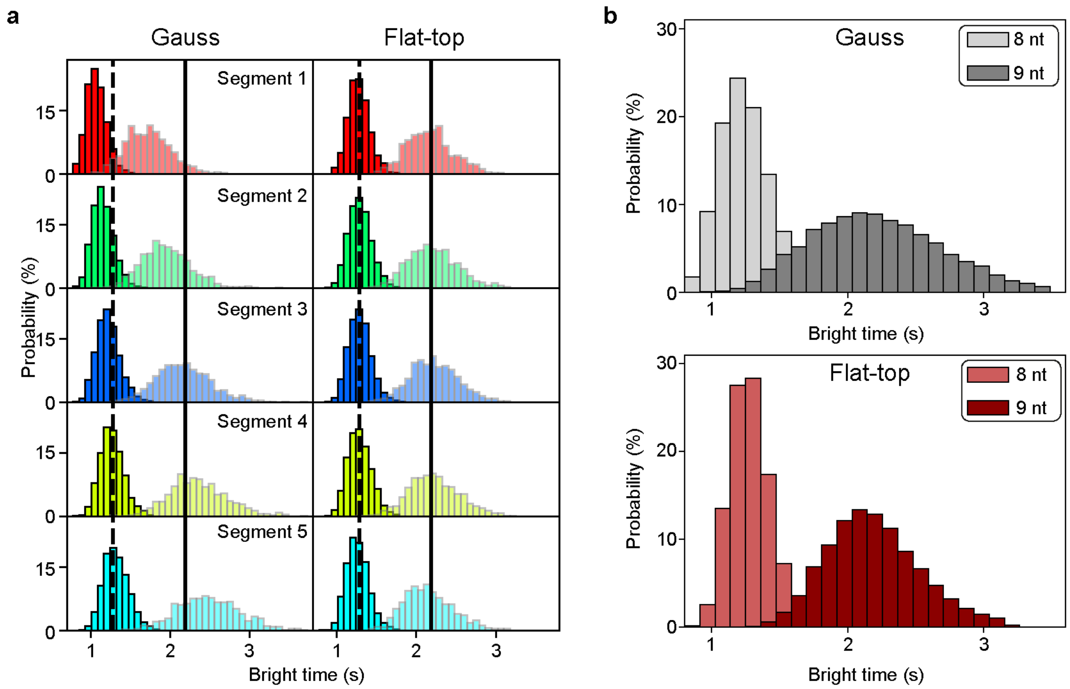

Supplementary Figure 3 | Bright time distributions for 8-nt vs. 9-nt docking site length. a Segmented bright time histograms for 20-nm-grids with 8-nt docking sites (opaque) and 9-nt docking site length (transparent) for Gauss and flat-top illumination. Mean bright times (over all segments) for flat-top illumination are indicated by dashed lines for 8-nt and solid lines for 9-nt. **b** Total bright time distributions for 8-nt vs 9-nt

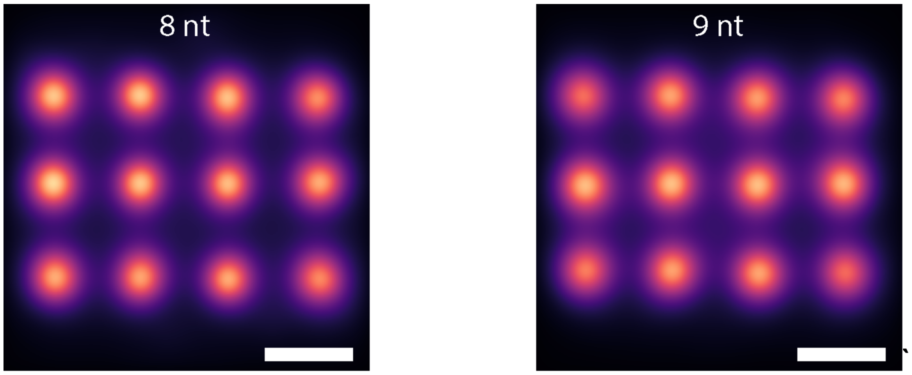

Supplementary Figure 4 | Averaged 20-nm-grid image for 8-nt vs. 9-nt docking sites for flat-top excitation. Over 8.000 DNA origami over the whole FOV for the flat-top data sets (8nt and 9nt docking strands) in Figure 2h and Supplementary Figure 5 were averaged and confirms that 20-nm resolution is conserved while origamis can be identified according to their bright times. Scale bars: 20 nm.

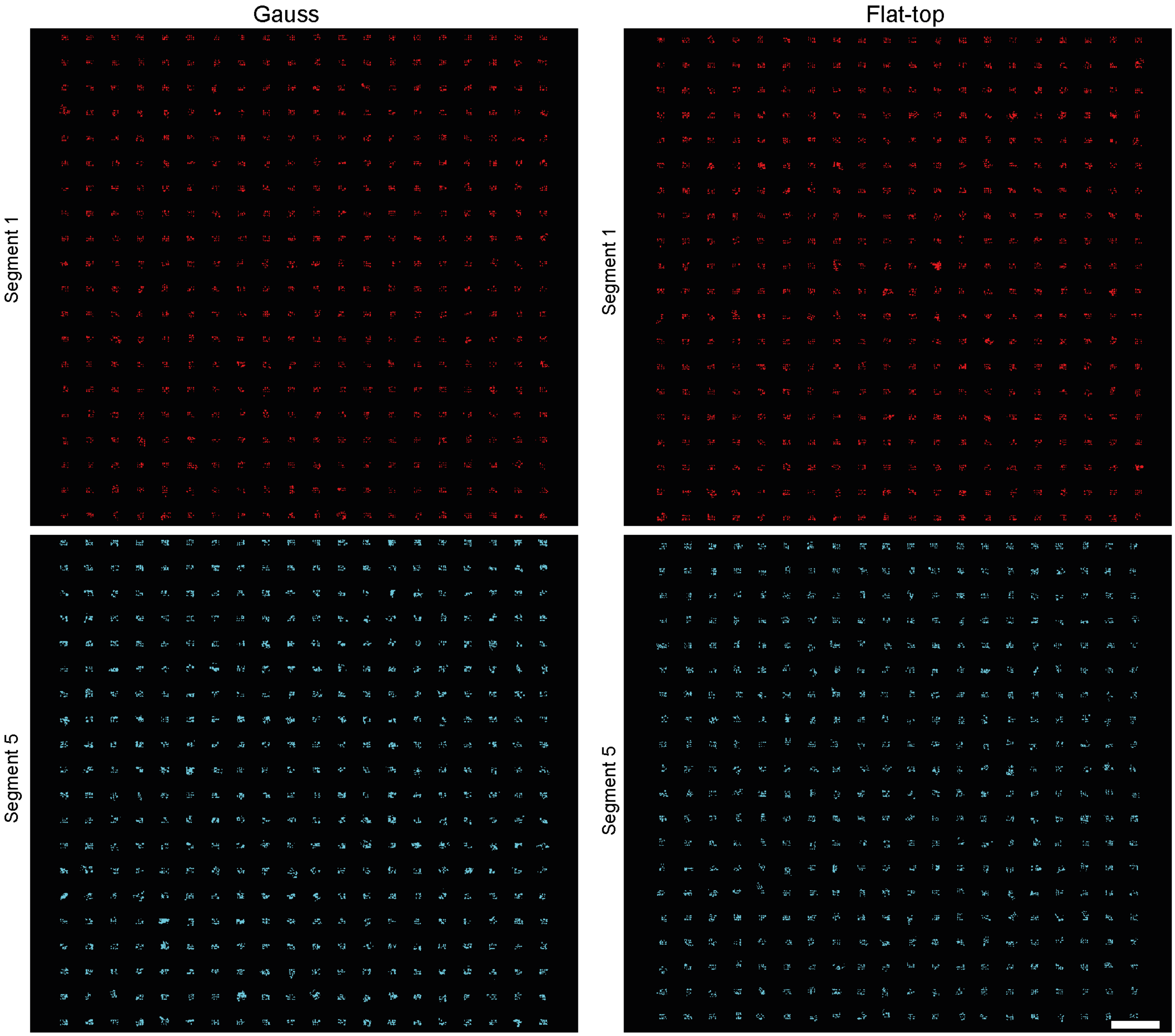

Supplementary Figure 5 | Individual origami images for averaging. Image showing 400 DNA origami structures extracted from segment 1 (red) and segment 5 (cyan) for DNA-PAINT images acquired with Gaussian (left) and flat-top illumination (right). In total more than 700 structures were used for averaging in Figure 3a. Scale bar: 500 nm.

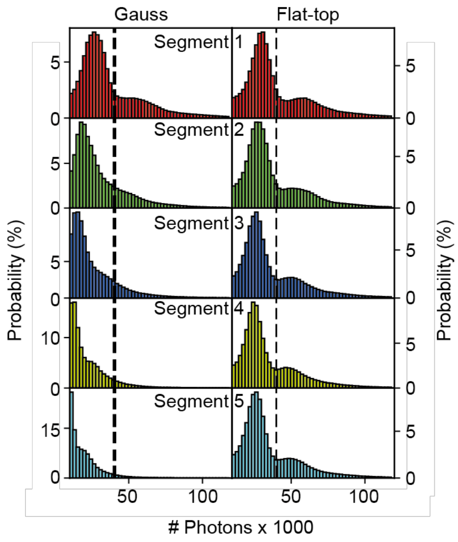

Supplementary Figure 6 | Segment-wise photon count distribution Gauss vs Flat-top.

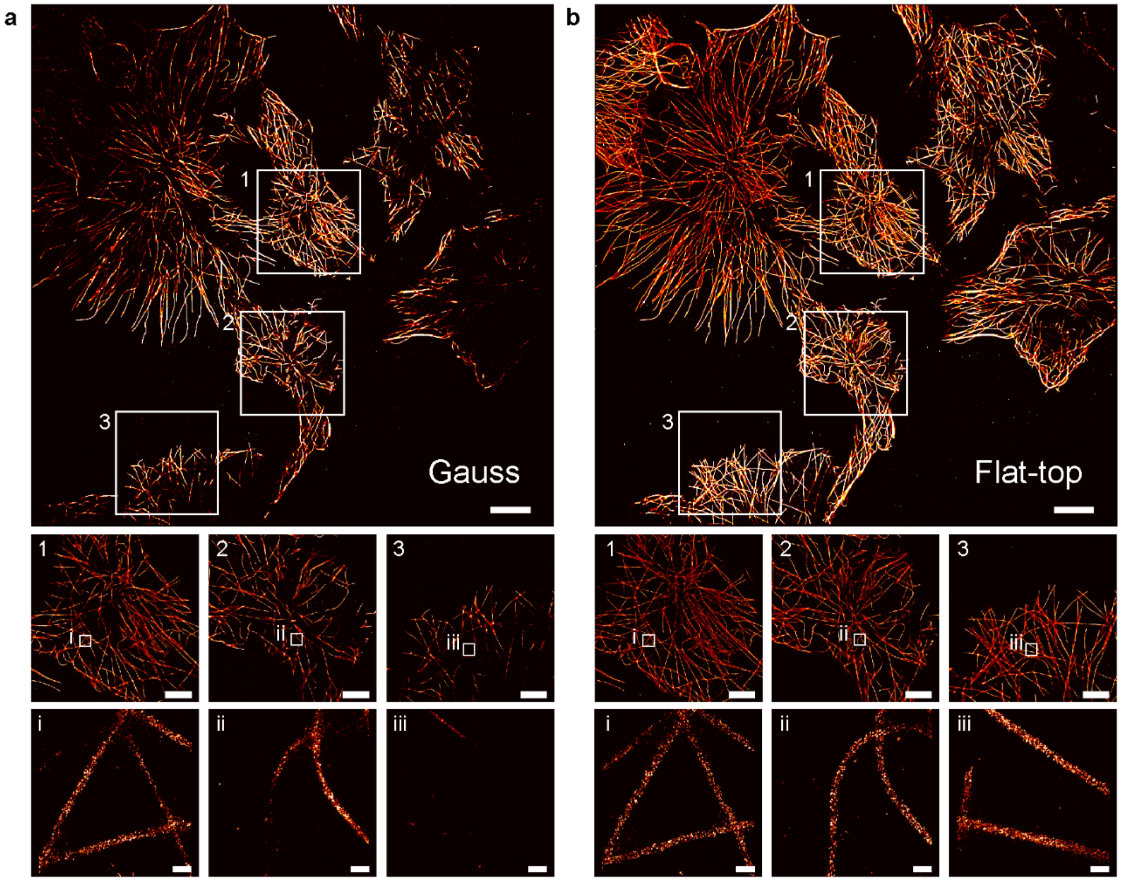

Supplementary Figure 7 | Uniform spot-detection efficiency for cellular DNA-PAINT imaging. a (top) Full camera chip (130×130 μm^2) DNA-PAINT image of the microtubule network in fixed COS-7 cells acquired using Gaussian illumination. (middle row) Three boxes highlighting the image quality in the center, intermediate and border region of the camera chip. (bottom row) Second-level zooms i)-iii) highlighting the inhomogeneous localization density over the whole image **b** Image of the same field of view as in **a** acquired with flat-top illumination profile for uniform localization density and image quality. Scale bars, 10 μm top images, 4 μm in middle row and 200 nm in bottom row.

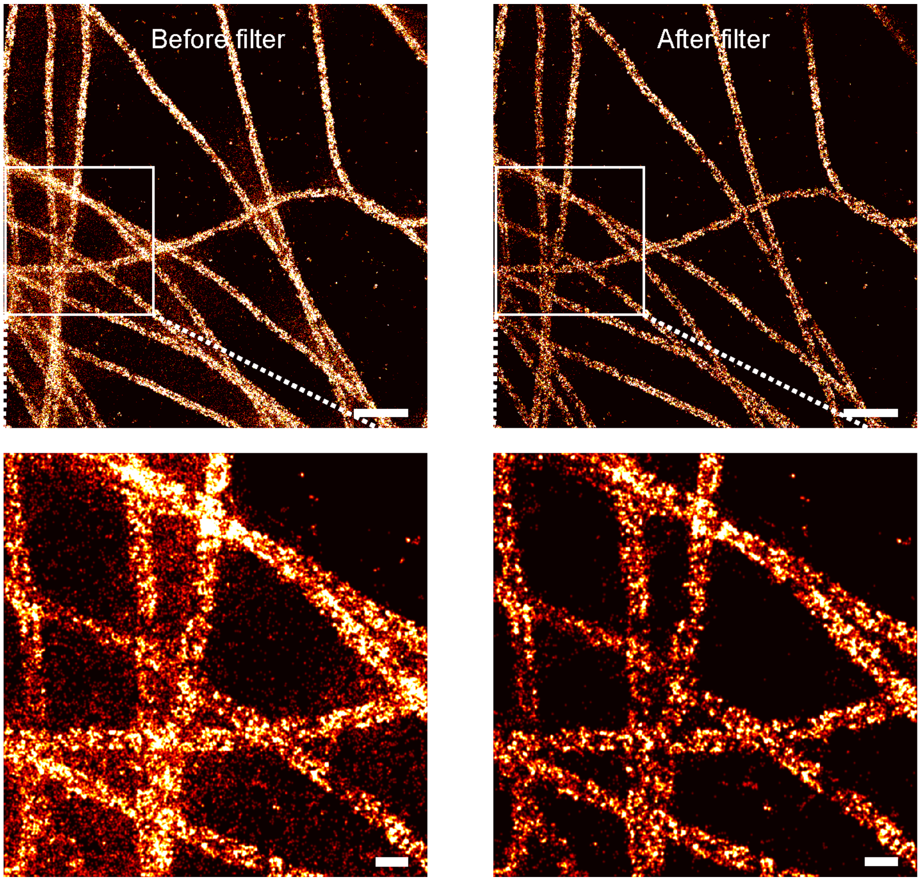

Supplementary Figure 8 | Mislocalization removal by multi-emitter filtering. Magnified region of cell image acquired under flat-top illumination (as in Figure 4b). Scale bars, top 500 nm, bottom 100 nm.

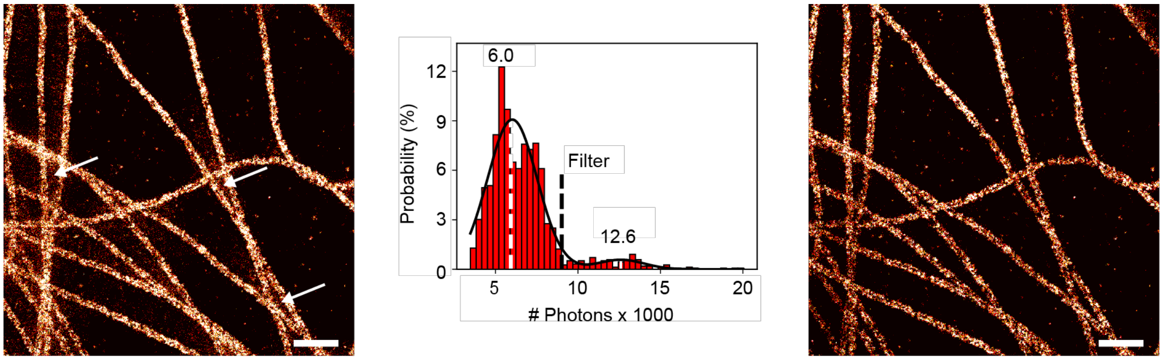

Supplementary Figure 9 | Mislocalization removal by multi-emitter filtering for Gaussian illumination. Magnified region of cell image acquired under Gaussian illumination (as in Figure 4b). Scale bars 500 nm.

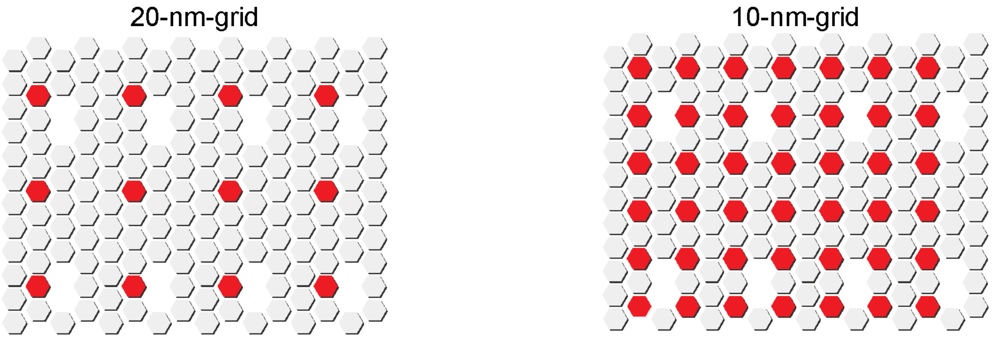

Supplementary Figure 10 | DNA origami grid designs. Picasso Design[1] schematic for 20- and 10-nm-grids. Red hexagons indicate extended staple strands for DNA-PAINT imaging. Missing hexagons indicate the position of extended staple strands on the opposite side, which are functionalized with biotin for surface immobilization.

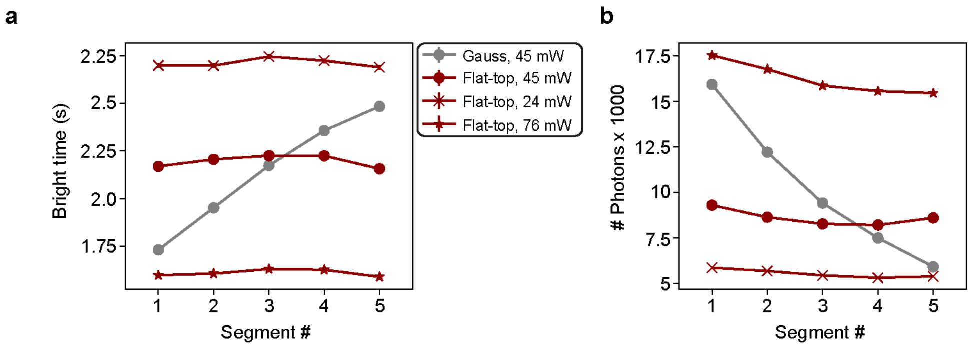

Supplementary Figure 11 | Laser power comparison Gauss vs. Flat-Top. a Mean bright time per segment for 9-nt 20-nm-grids **b** Mean number of detected photons per localization with respect to each segment for same data sets as in **a**.

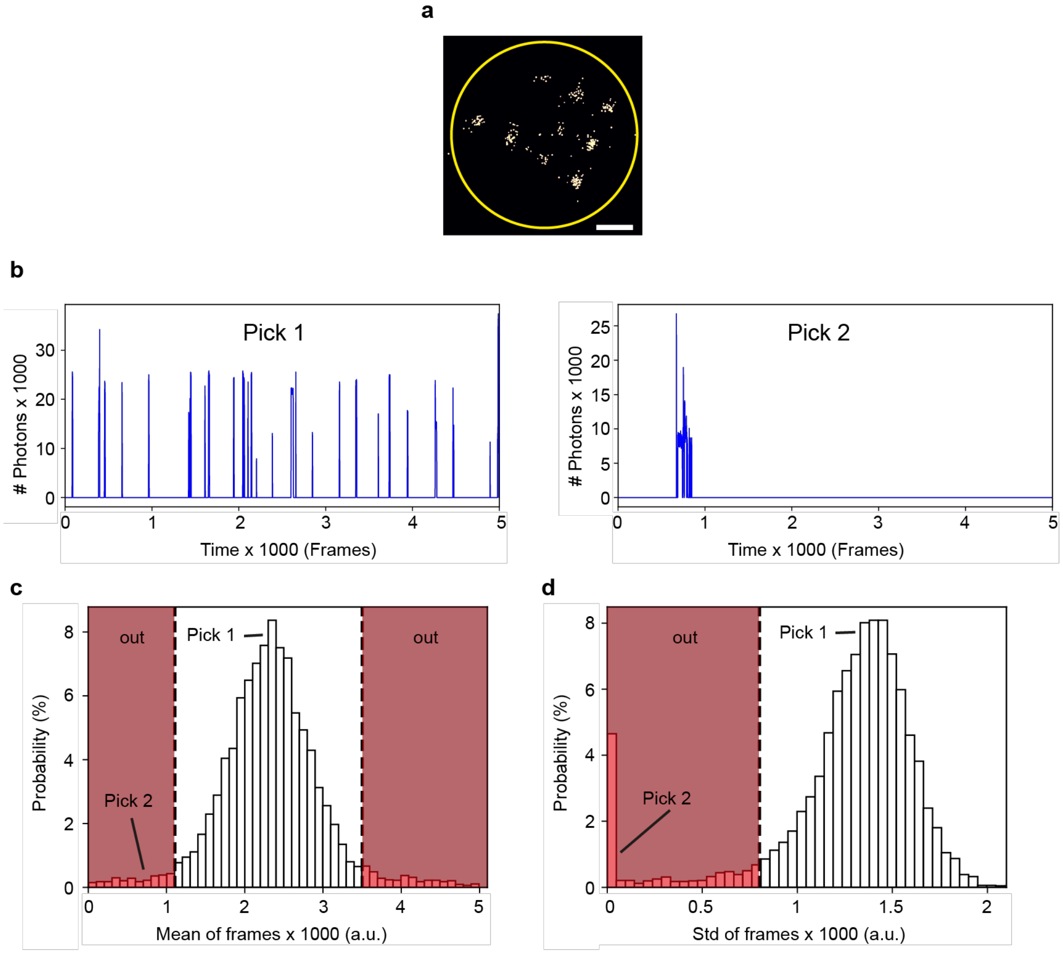

Supplementary Figure 12 | Filtering by temporal distribution of localizations. a Exemplary auto-selected ROI (yellow circle) of a 20-nm grid in the rendered super-resolution image **b** Typical intensity traces from picks showing repetitive (left) and non-repetitive blinking behavior (right) **c** Exemplary distribution of the mean (localization) frame of all auto-selected picks and **d** the respective distribution for the standard deviation. Values corresponding to picks shown in **b** are indicated. Red areas in both **c** and **d** are discarded before further analysis.

Supplementary Table 1 | List of core staples

Position	Name	Sequence
A1	21[32]23[31]BLK	TTTTCACTCAAAGGGCGAAAAACCATCACC
B1	23[32]22[48]BLK	CAAATCAAGTTTTTTGGGGTCGAAACGTGGA
C1	21[56]23[63]BLK	AGCTGATTGCCCTTCAGAGTCCACTATTAAAGGGTGCCGT
D1	23[64]22[80]BLK	AAAGCACTAAATCGGAACCCTAATCCAGTT
E1	21[96]23[95]BLK	AGCAAGCGTAGGGTTGAGTGTTGTAGGGAGCC
F1	23[96]22[112]BLK	CCCGATTTAGAGCTTGACGGGGAAAAAGAATA
G1	21[120]23[127]BLK	CCCAGCAGGCGAAAAATCCCTTATAAATCAAGCCGGCG
H1	21[160]22[144]BLK	TCAATATCGAACCTCAAATATCAATTCCGAAA
I1	23[128]23[159]BLK	AACGTGGCGAGAAAGGAAGGGAAACCAGTAA
J1	23[160]22[176]BLK	TAAAAGGGACATTCTGGCCAACAAAGCATC
K1	21[184]23[191]BLK	TCAACAGTTGAAAGGAGCAAATGAAAAATCTAGAGATAGA
L1	23[192]22[208]BLK	ACCCTTCTGACCTGAAAGCGTAAGACGCTGAG
M1	21[224]23[223]BLK	CTTTAGGGCCTGCAACAGTGCCAATACGTG
N1	23[224]22[240]BLK	GCACAGACAATATTTTTGAATGGGGTCAGTA
O1	21[248]23[255]BLK	AGATTAGAGCCGTCAAAAAACAGAGGTGAGGCCTATTAGT
P1	23[256]22[272]BLK	CTTTAATGCGCGAACTGATAGCCCCACCAG
A2	19[32]21[31]BLK	GTCGACTTCGGCCAACGCGCGGGGTTTTTC
B2	22[47]20[48]BLK	CTCCAACGCAGTGAGACGGGCAACCAGCTGCA
D2	22[79]20[80]BLK	TGGAACAACCGCCTGGCCCTGAGGCCCGCT
E2	19[96]21[95]BLK	CTGTGTGATTGCGTTGCGCTCACTAGAGTTGC
F2	22[111]20[112]BLK	GCCCGAGAGTCCACGCTGGTTTGCAGCTAACT
H2	19[160]20[144]BLK	GCAATTCACATATTCCTGATTATCAAAGTGTA
I2	22[143]21[159]BLK	TCGGCAAATCCTGTTTGATGGTGGACCCTCAA
J2	22[175]20[176]BLK	ACCTTGCTTGGTCAGTTGGCAAAGAGCGGA
L2	22[207]20[208]BLK	AGCCAGCAATTGAGGAAGGTTATCATCATTTT
M2	19[224]21[223]BLK	CTACCATAGTTTGAGTAACATTTAAAATAT
N2	22[239]20[240]BLK	TTAACACCAGCACTAACAACTAATCGTTATTA
P2	22[271]20[272]BLK	CAGAAGATTAGATAATACATTTGTCGACAA
A3	17[32]19[31]BLK	TGCATCTTTCCCAGTCACGACGGCCTGCAG
B3	20[47]18[48]BLK	TTAATGAACTAGAGGATCCCCGGGGGGTAACG
D3	20[79]18[80]BLK	TTCCAGTCGTAATCATGGTCATAAAAGGGG
E3	17[96]19[95]BLK	GCTTTCCGATTACGCCAGCTGGCGGCTGTTTC
F3	20[111]18[112]BLK	CACATTAAAATTGTTATCCGCTCATGCGGGCC
H3	17[160]18[144]BLK	AGAAAACAAAGAAGATGATGAAACAGGCTGCG
I3	20[143]19[159]BLK	AAGCCTGGTACGAGCCGGAAGCATAGATGATG
J3	20[175]18[176]BLK	ATTATCATTCAATATAATCCTGACAATTAC

L3	20[207]18[208]BLK	GCGGAACATCTGAATAATGGAAGGTACAAAAT
M3	17[224]19[223]BLK	CATAAATCTTTGAATACCAAGTGTTAGAAC
N3	20[239]18[240]BLK	ATTTTAAAATCAAAATTATTTGCACGGATTCG
P3	20[271]18[272]BLK	CTCGTATTAGAAATTGCGTAGATACAGTAC
A4	15[32]17[31]BLK	TAATCAGCGGATTGACCGTAATCGTAACCG
B4	18[47]16[48]BLK	CCAGGGTTGCCAGTTTGAGGGGACCCGTGGGA
C4	15[64]18[64]BLK	GTATAAGCCAACCCGTCGGATTCTGACGACAGTATCGGCCGCAAGGCG
D4	18[79]16[80]BLK	GATGTGCTTCAGGAAGATCGCACAATGTGA
E4	15[96]17[95]BLK	ATATTTTGGCTTTCATCAACATTATCCAGCCA
F4	18[111]16[112]BLK	TCTTCGCTGCACCGCTTCTGGTGCGGCCTTCC
G4	15[128]18[128]BLK	TAAATCAAAATAATTCGCGTCTCGGAAACCAGGCAAAGGGAAGG
H4	15[160]16[144]BLK	ATCGCAAGTATGTAAATGCTGATGATAGGAAC
I4	18[143]17[159]BLK	CAACTGTTGCGCCATTCGCCATTCAAACATCA
J4	18[175]16[176]BLK	CTGAGCAAAAATTAATTACATTTTGGGTTA
K4	15[192]18[192]BLK	TCAAATATAACCTCCGGCTTAGGTAACAATTTCATTTGAAGGCGAATT
L4	18[207]16[208]BLK	CGCGCAGATTACCTTTTTAATGGGAGAGACT
M4	15[224]17[223]BLK	CCTAAATCAAAATCATAGGTCTAAACAGTA
N4	18[239]16[240]BLK	CCTGATTGCAATATATGTGAGTGATCAATAGT
O4	15[256]18[256]BLK	GTGATAAAAAGACGCTGAGAAGAGATAACCTTGCTTCTGTTCGGGAGA
P4	18[271]16[272]BLK	CTTTTACAAAATCGTCGCTATTAGCGATAG
A5	13[32]15[31]BLK	AACGCAAAATCGATGAACGGTACCGGTTGA
B5	16[47]14[48]BLK	ACAAACGGAAAAGCCCCAAAAACACTGGAGCA
C5	13[64]15[63]BLK	TATATTTTGTCATTGCCTGAGAGTGGAAGATT
D5	16[79]14[80]BLK	GCGAGTAAAAATATTTAAATTGTTACAAAG
E5	13[96]15[95]BLK	TAGGTAAACTATTTTTGAGAGATCAAACGTTA
F5	16[111]14[112]BLK	TGTAGCCATTAAAATTCGCATTAAATGCCGGA
G5	13[128]15[127]BLK	GAGACAGCTAGCTGATAAATTAATTTTTGT
H5	13[160]14[144]BLK	GTAATAAGTTAGGCAGAGGCATTTATGATATT
I5	16[143]15[159]BLK	GCCATCAAGCTCATTTTTTAACCACAAATCCA
J5	16[175]14[176]BLK	TATAACTAACAAAGAACGCGAGAACGCCAA
K5	13[192]15[191]BLK	GTAAAGTAATCGCCATATTTAACAAAACTTTT
L5	16[207]14[208]BLK	ACCTTTTTATTTTAGTTAATTTCATAGGGCTT
M5	13[224]15[223]BLK	ACAACATGCCAACGCTCAACAGTCTTCTGA
N5	16[239]14[240]BLK	GAATTTATTTAATGGTTTGAAATATTCTTACC
O5	13[256]15[255]BLK	GTTTATCAATATGCGTTATACAAACCGACCGT
P5	16[271]14[272]BLK	CTTAGATTTAAGGCGTTAAATAAAGCCTGT
A6	11[32]13[31]BLK	AACAGTTTTGTACCAAAAACATTTTATTTC
B6	14[47]12[48]BLK	AACAAGAGGGATAAAAATTTTTAGCATAAAGC

C6	11[64]13[63]BLK	GATTTAGTCAATAAAGCCTCAGAGAACCCTCA
D6	14[79]12[80]BLK	GCTATCAGAAATGCAATGCCTGAATTAGCA
E6	11[96]13[95]BLK	AATGGTCAACAGGCAAGGCAAAGAGTAATGTG
F6	14[111]12[112]BLK	GAGGGTAGGATTCAAAAGGGTGAGACATCCAA
G6	11[128]13[127]BLK	TTTGGGGATAGTAGTAGCATTAAAAGGCCG
H6	11[160]12[144]BLK	CCAATAGCTCATCGTAGGAATCATGGCATCAA
I6	14[143]13[159]BLK	CAACCGTTTCAAATCACCATCAATTCGAGCCA
J6	14[175]12[176]BLK	CATGTAATAGAATATAAAGTACCAAGCCGT
K6	11[192]13[191]BLK	TATCCGGTCTCATCGAGAACAAGCGACAAAAG
L6	14[207]12[208]BLK	AATTGAGAATTCTGTCCAGACGACTAAACCAA
M6	11[224]13[223]BLK	GCGAACCTCCAAGAACGGGTATGACAATAA
N6	14[239]12[240]BLK	AGTATAAAGTTCAGCTAATGCAGATGTCTTTC
O6	11[256]13[255]BLK	GCCTTAAACCAATCAATAATCGGCACGCGCCT
P6	14[271]12[272]BLK	TTAGTATCACAATAGATAAGTCCACGAGCA
A7	9[32]11[31]BLK	TTTACCCCAACATGTTTTAAATTTCCATAT
B7	12[47]10[48]BLK	TAAATCGGGATTCCCAATTCTGCGATATAATG
C7	9[64]11[63]BLK	CGGATTGCAGAGCTTAATTGCTGAAACGAGTA
D7	12[79]10[80]BLK	AAATTAAGTTGACCATTAGATACTTTTGCG
E7	9[96]11[95]BLK	CGAAAGACTTTGATAAGAGGTCATATTTCGCA
F7	12[111]10[112]BLK	TAAATCATATAACCTGTTTAGCTAACCTTTAA
G7	9[128]11[127]BLK	GCTTCAATCAGGATTAGAGAGTTATTTTCA
H7	9[160]10[144]BLK	AGAGAGAAAAAAATGAAAATAGCAAGCAAACT
I7	12[143]11[159]BLK	TTCTACTACGCGAGCTGAAAAGGTTACCGCGC
J7	12[175]10[176]BLK	TTTTATTTAAGCAAATCAGATATTTTTTGT
K7	9[192]11[191]BLK	TTAGACGGCCAAATAAGAAACGATAGAAGGCT
L7	12[207]10[208]BLK	GTACCGCAATTCTAAGAACGCGAGTATTATTT
M7	9[224]11[223]BLK	AAAGTCACAAAATAAACAGCCAGCGTTTTA
N7	12[239]10[240]BLK	CTTATCATTCCCGACTTGCGGGAGCCTAATTT
O7	9[256]11[255]BLK	GAGAGATAGAGCGTCTTTCCAGAGGTTTTGAA
P7	12[271]10[272]BLK	TGTAGAAATCAAGATTAGTTGCTCTTACCA
A8	7[32]9[31]BLK	TTTAGGACAAATGCTTTAAACAATCAGGTC
B8	10[47]8[48]BLK	CTGTAGCTTGACTATTATAGTCAGTTCATTGA
C8	7[56]9[63]BLK	ATGCAGATACATAACGGGAATCGTCATAAATAAAGCAAAG
D8	10[79]8[80]BLK	GATGGCTTATCAAAAAGATTAAGAGCGTCC
E8	7[96]9[95]BLK	TAAGAGCAAATGTTTAGACTGGATAGGAAGCC
F8	10[111]8[112]BLK	TTGCTCCTTTCAAATATCGCGTTTGAGGGGGT
G8	7[120]9[127]BLK	CGTTTACCAGACGACAAAGAAGTTTTGCCATAATTCGA
H8	7[160]8[144]BLK	TTATTACGAAGAACTGGCATGATTGCGAGAGG

I8	10[143]9[159]BLK	CCAACAGGAGCGAACCAGACCGGAGCCTTTAC
J8	10[175]8[176]BLK	TTAACGTCTAACATAAAAACAGGTAACGGA
K8	7[184]9[191]BLK	CGTAGAAAATACATACCGAGGAAACGCAATAAGAAGCGCA
L8	10[207]8[208]BLK	ATCCCAATGAGAATTAACTGAACAGTTACCAG
M8	7[224]9[223]BLK	AACGCAAAGATAGCCGAACAAACCCTGAAC
N8	10[239]8[240]BLK	GCCAGTTAGAGGGTAATTGAGCGCTTTAAGAA
O8	7[248]9[255]BLK	GTTTATTTTGTCACAATCTTACCGAAGCCCTTTAATATCA
P8	10[271]8[272]BLK	ACGCTAACACCCACAAGAATTGAAAATAGC
A9	5[32]7[31]BLK	CATCAAGTAAAACGAACTAACGAGTTGAGA
B9	8[47]6[48]BLK	ATCCCCCTATACCACATTCAACTAGAAAAATC
D9	8[79]6[80]BLK	AATACTGCCCAAAAGGAATTACGTGGCTCA
E9	5[96]7[95]BLK	TCATTCAGATGCGATTTTAAGAACAGGCATAG
F9	8[111]6[112]BLK	AATAGTAAACACTATCATAACCCTCATTGTGA
H9	5[160]6[144]BLK	GCAAGGCCTCACCAGTAGCACCATGGGCTTGA
I9	8[143]7[159]BLK	CTTTTGCAGATAAAAACCAAAATAAAGACTCC
J9	8[175]6[176]BLK	ATACCCAACAGTATGTTAGCAAATTAGAGC
L9	8[207]6[208]BLK	AAGGAAACATAAAGGTGGCAACATTATCACCG
M9	5[224]7[223]BLK	TCAAGTTTCATTAAAGGTGAATATAAAAGA
N9	8[239]6[240]BLK	AAGTAAGCAGACACCACGGAATAATATTGACG
P9	8[271]6[272]BLK	AATAGCTATCAATAGAAAATTCAACATTCA
A10	3[32]5[31]BLK	AATACGTTTGAAAGAGGACAGACTGACCTT
B10	6[47]4[48]BLK	TACGTTAAAGTAATCTTGACAAGAACCGAACT
D10	6[79]4[80]BLK	TTATACCACCAAATCAACGTAACGAACGAG
E10	3[96]5[95]BLK	ACACTCATCCATGTTACTTAGCCGAAAGCTGC
F10	6[111]4[112]BLK	ATTACCTTTGAATAAGGCTTGCCCAAATCCGC
H10	3[160]4[144]BLK	TTGACAGGCCACCACCAGAGCCGCGATTTGTA
I10	6[143]5[159]BLK	GATGGTTTGAACGAGTAGTAAATTTACCATTA
J10	6[175]4[176]BLK	CAGCAAAAGGAAACGTCACCAATGAGCCGC
L10	6[207]4[208]BLK	TCACCGACGCACCGTAATCAGTAGCAGAACCG
M10	3[224]5[223]BLK	TTAAAGCCAGAGCCGCCACCCTCGACAGAA
N10	6[239]4[240]BLK	GAAATTATTGCCTTTAGCGTCAGACCGGAACC
P10	6[271]4[272]BLK	ACCGATTGTCGGCATTTTCGGTCATAATCA
A11	1[32]3[31]BLK	AGGCTCCAGAGGCTTTGAGGACACGGGTAA
B11	4[47]2[48]BLK	GACCAACTAATGCCACTACGAAGGGGGTAGCA
C11	1[64]4[64]BLK	TTTATCAGGACAGCATCGGAACGACACCAACCTAAAACGAGGTCAATC
D11	4[79]2[80]BLK	GCGCAGACAAGAGGCAAAAGAATCCCTCAG
E11	1[96]3[95]BLK	AAACAGCTTTTTGCGGGATCGTCAACACTAAA
F11	4[111]2[112]BLK	GACCTGCTCTTTGACCCCCAGCGAGGGAGTTA

G11	1[128]4[128]BLK	TGACAACTCGCTGAGGCTTGCATTATACCAAGCGCGATGATAAA
H11	1[160]2[144]BLK	TTAGGATTGGCTGAGACTCCTCAATAACCGAT
I11	4[143]3[159]BLK	TCATCGCCAACAAAGTACAACGGACGCCAGCA
J11	4[175]2[176]BLK	CACCAGAAAGGTTGAGGCAGGTCATGAAAG
K11	1[192]4[192]BLK	GCGGATAACCTATTATTCTGAAACAGACGATTGGCCTTGAAGAGCCAC
L11	4[207]2[208]BLK	CCACCCTCTATTCACAAACAAATACCTGCCTA
M11	1[224]3[223]BLK	GTATAGCAAACAGTTAATGCCCAATCCTCA
N11	4[239]2[240]BLK	GCCTCCCTCAGAATGGAAAGCGCAGTAACAGT
O11	1[256]4[256]BLK	CAGGAGGTGGGGTCAGTGCCTTGAGTCTCTGAATTTACCGGGAACCAG
P11	4[271]2[272]BLK	AAATCACCTTCCAGTAAGCGTCAGTAATAA
A12	0[47]1[31]BLK	AGAAAGGAACAACTAAAGGAATTCAAAAAAA
B12	2[47]0[48]BLK	ACGGCTACAAAAGGAGCCTTTAATGTGAGAAT
C12	0[79]1[63]BLK	ACAACTTTCAACAGTTTCAGCGGATGTATCGG
D12	2[79]0[80]BLK	CAGCGAAACTTGCTTTCGAGGTGTTGCTAA
E12	0[111]1[95]BLK	TAAATGAATTTTCTGTATGGGATTAATTTCTT
F12	2[111]0[112]BLK	AAGGCCGCTGATACCGATAGTTGCGACGTTAG
G12	0[143]1[127]BLK	TCTAAAGTTTTGTCGTCTTTCCAGCCGACAA
H12	0[175]0[144]BLK	TCCACAGACAGCCCTCATAGTTAGCGTAACGA
I12	2[143]1[159]BLK	ATATTCGGAACCATCGCCCACGCAGAGAAGGA
J12	2[175]0[176]BLK	TATTAAGAAGCGGGGTTTTGCTCGTAGCAT
K12	0[207]1[191]BLK	TCACCAGTACAAACTACAACGCCTAGTACCAG
L12	2[207]0[208]BLK	TTTCGGAAGTGCCGTCGAGAGGGTGAGTTTCG
M12	0[239]1[223]BLK	AGGAACCCATGTACCGTAACACTTGATATAA
N12	2[239]0[240]BLK	GCCCGTATCCGGAATAGGTGTATCAGCCCAAT
O12	0[271]1[255]BLK	CCACCCTCATTTTCAGGGATAGCAACCGTACT
P12	2[271]0[272]BLK	GTTTTAACTTAGTACCGCCACCCAGAGCCA

Supplementary Table 2 | List of biotinylated staples

No	Pos	Name	Sequence	Mod
1	C02	18[63]20[56]BIOTIN	ATTAAGTTTACCGAGCTCGAATTCGGGAAACCTGTCGTGC	5'-BT
2	C09	4[63]6[56]BIOTIN	ATAAGGGAACCGGATATTCATTACGTCAGGACGTTGGGAA	5'-BT
3	G02	18[127]20[120]BIOTIN	GCGATCGGCAATTCCACACAACAGGTGCCTAATGAGTG	5'-BT
4	G09	4[127]6[120]BIOTIN	TTGTGTCGTGACGAGAAACACCAAATTTCAACTTTAAT	5'-BT
5	K02	18[191]20[184]BIOTIN	ATTCATTTTTGTTTGGATTATACTAAGAAACCACCAGAAG	5'-BT
6	K09	4[191]6[184]BIOTIN	CACCCTCAGAAACCATCGATAGCATTGAGCCATTTGGGAA	5'-BT

7	O02	18[255]20[248]BIOTIN	AACAATAACGTAAAACAGAAATAAAAATCCTTTGCCCGAA	5'-BT
8	O09	4[255]6[248]BIOTIN	AGCCACCACTGTAGCGCGTTTTCAAGGGAGGGAAGGTAAA	5'-BT

Supplementary Table 3 | Imaging parameters

Figure	Sample	Origami dilution (after PEG purification)	Docking site sequence	Imager concentration (nM)	Imaging Buffer	Laser power before objective (mW)	Exposure time (ms)	Frames	Binning
1	20-nm-grid	1:1	P1 (9 nt)	4	B	Gauss: 1.5<br>Flat-top: 2.4	200	200	16x16
2c,d,e<br>SI_Fig. 2	20-nm-grid	1:80	P1 (9 nt)	4	B	Gauss: 45<br>Flat-top: 77	200	5,000	2x2
2f,g<br>2h (9 nt)<br>SI_Fig. 3,4 (9 nt)	20-nm grid	1:80	P1 (9 nt)	4	B	Gauss: 45<br>Flat-top: 45	200	13,000	2x2
2h (8 nt)<br>SI_Fig. 3,4 (8 nt)	20-nm-grid	1:80	P1 (8 nt)	4	B	Gauss: 45<br>Flat-top: 45	200	13,000	2x2
3a,b,c<br>SI_Fig. 5	20-nm-grid	1:80	P1 (9 nt)	4	B	Gauss: 45<br>Flat-top: 77	200	5,000	2x2
3d	10-nm-grid	1:100	P1 (9 nt)	0.5	1× B<br>1× Trolox<br>1× PCA<br>1× PCD	Flat-top: 77	200	25,000	2x2
3e,f<br>SI_Fig. 6	20-nm-grid	1:80	P1 (9 nt)	20	B	Gauss: 78<br>Flat-top: 132	200	10,000	2x2
4<br>SI_Fig. 8,9	COS-7	-	P1 (8 nt)	0.15	1× C<br>1× Trolox<br>1× PCA<br>1× PCD	Gauss: 42<br>Flat-top: 25	150	60,000	2x2
SI_Fig. 7	COS-7	-	P1 (9 nt)	0.2	1× C<br>1× Trolox<br>1× PCA<br>1× PCD	Gauss: 90<br>Flat-top: 153	150	13,000	2x2
SI_Fig. 11	20-nm-grid	1:80	P1 (9 nt)	4	B	Gauss: 45<br>Flat-top: 24, 45, 76	200	5,000	2x2

Supplementary Table 4 | Used DNA-PAINT sequences

**Shortname (docking site length)**	**Docking sequence**	**Imager sequence**	**Experiment**
P1 (9 nt)	TT ATACATCTA	CTAGATGTAT-Cy3b	All except the ones stated below
P1 (8 nt)	TT ATACATCT	CTAGATGTAT-Cy3b	Fig. 2h<br>SI_Fig. 6
P1 (10 nt)	TT ATACATCTAG	AGATGTAT-Cy3b	Fig. 4<br>SI_Fig. 8

Supplementary references

1. Schnitzbauer, J., Strauss, M. T., Schlichthaerle, T., Schueder, F. & Jungmann, R. Super-resolution microscopy with DNA-PAINT. *Nat. Protoc.* **12**, 1198 (2017).
